# Importance of Details

**Published:** 1902-12

**Authors:** W. M. Whitlock


### IMPORTANCE OF DETAILS.[1]

BY DR. W. M. WHITLOCK.

PROBABLY there is nothing relating to our profession to which so much emphasis has been given as the importance of details. Every dental operation must be performed with this thought always in mind, and every dental operation is successful exactly in proportion to the amount of care given each step. We cannot say that this carefulness of detail is more important in one class of operations than another, but nowhere does hasty, incomplete, careless work make itself so painfully felt as in operations in the roots of teeth. Every crown needs a good root, and the best root is a live one in which the pulp is healthy and normal, but if the pulp has become inflamed, perhaps only irritated, comfort may demand its extirpation. The pulp may be dead and not removed, and hence a source of danger. But if, from whatever cause, it becomes necessary to open a tooth, treat and fill the canals, let that operation be

---

[1] Read at a meeting of The New York Institute of Stomatology, June 3, 1902.

done, step by step, with the minutest care, lest the last state of that tooth be no better, if not worse, than the first. With this thought before us, let me select one step in that operation—"the opening of root canals"—for a few words regarding my methods of procedure. Having made our diagnosis and having decided that the pulp must be lost or that the tooth is dead, we should allow nothing to hinder or prevent our entering and cleansing every canal, and if, in so doing, it becomes necessary to sacrifice tooth-structure, we should unhesitatingly do so, for certainly the crown of the tooth is of no value without the roots, yet we must be careful lest we so weaken the tooth as to increase the danger of its splitting through both crown and root. While deprecating the unnecessary cutting away of tooth-structure, with a perfect knowledge of the location of the canals of normal teeth and a knowledge of their probable location from the outline and conformity of the visible portion of the tooth, the canals can all be entered with a minimum amount of cutting. An excellent way of attaining this knowledge is by a study of teeth out of the mouth, by noticing their shape and the general relation of the roots to the crown.

It is not my intention to give a detailed account of my method of opening each individual tooth, but it might not be amiss to mention a few points that have been of inestimable value to me. In speaking of difficulties encountered in opening teeth, it is the multi-rooted teeth, the molars and bicuspids, that are generally to be considered. Of course, the reason for this is obvious, as the liability of small, abnormal, tortuous canals is much greater in these teeth.

In opening molar teeth it is my practice always to ignore a posterior or buccal cavity. If the tooth presents a compound anterior cavity, this can be utilized, otherwise an opening should be made through the anterior sulcus. It is desirable, in bicuspid teeth, to avoid going down through the centre if possible, using an anterior or posterior cavity, lest the tooth be too greatly weakened. In entering, extreme care must be taken not to mar the floor of the pulp-chamber, for its natural conformation is our most valuable guide to the location of the canals. Without enlarging the external opening to any great extent, the inner end can be bevelled to include the whole roof of the chamber. This is best accomplished by a round bur of medium size, either straight or right-angled, as the case requires.

In entering the anterior sulcus of a normal superior molar the drill enters the pulp-chamber very close to the centre; that is, at a point midway between the three canals; then by hooking the bur under the palatine edge of the roof of the chamber and pulling up, always being careful not to mar the bottom of the chamber, that portion over the palatine canal may be removed so that the canal can very readily be entered with the broach. The same method is used buccally and a little posteriorly for the posterior-buccal canal, while, especially in extreme cases, considerable anterior cutting is required to enter the anterior-buccal canal.

Entering the pulp-chamber from the anterior sulcus of a lower molar finds us, in nearly all cases, directly over the anterior-lingual canal. From this point it is necessary to bevel posteriorly and somewhat laterally to a considerable extent to enable us to enter the posterior canal freely, while the anterior-buccal canal will be found usually to be, if anything, somewhat anterior to the lingual. The possibility of a fourth canal must always be borne in mind in opening these lower molars, there being often, instead of one large posterior canal, a buccal and a lingual canal posteriorly as well as anteriorly.

Here again let me emphasize the great advantage to be gained in not destroying the natural formation of the floor of the chamber, for the canals are at the bottom of a funnel-shaped depression, which is an invaluable guide to locating them, and in cutting these away we add very much to our troubles. It is also a great mistake to try to enter the canals with an engine-bur or a large stiff instrument of any kind, for in doing so the danger of going through the side of the canal, or, at least, of making a shoulder that will prevent free access, is very great. What I have said thus far has reference, principally, to normal teeth. There are some abnormalities with which we are familiar and many more with which we are not at all familiar but which require watchfulness and care in observing and filling what we see and not what we expect to find. There are two that are somewhat common. One that I have found, and a very puzzling one, is pulp-stone, of which I have samples here. They are found in teeth that are otherwise normal and can be broken up in the pulp-chamber or taken out intact. The other abnormality is to be found in the upper second molar, which frequently presents a large palatine and buccal canal, with a very small one between them, which, if we enter at all, will be but a little way.

Not of the least importance are the instruments to be used, and to which I wish to call your special attention.

First, a smooth, round, piano-wire broach, used for locating, examining, and measuring canals. Normal teeth usually measure about three-quarters of an inch in length. It is well to measure each tooth through the large canal, and make a note of any variation from this normal length. Indeed, you may measure each root. It is important to have these smooth broaches marked with a bead of shellac, so that we will know how far we are going.

Second, a four-sided, square, piano-wire broach, for reaming and enlarging the canal. These broaches are very tough, and with moderate care will not break. They will even twist into a spiral form first, when, of course, it is better to discard them. They are all pliable, conforming readily to an ordinarily crooked canal. By letting the instrument pass up until it binds, and then freeing it a little and turning it back and forth between the fingers, the fine canals can in a very short time be enlarged sufficiently to be readily cleansed and filled.

Third, a small, three-sided finger-reamer, such as suggested by Dr. Howe, for enlarging the entrance to each canal to aid in dressing and filling.

A Donaldson canal-cleanser is a very valuable instrument, not only for removing a pulp intact, but, in conjunction with pyrozone, for removing the disorganized remains of the pulp in a tooth already dead, and for scraping the sides of such a canal. I have found sizes No. 5 and medium to be the most serviceable.

In many dead teeth that have been long neglected the carious process has extended to the canals. This leathery decay can best be removed by means of a smooth wire broach, such a one as has already been described, the end flattened and turned at nearly right angles and sharpened as a hoe. Cleansing the canals of such teeth is not difficult, as they have already been enlarged by decay.

During all this instrumentation the broaches and cleansers are constantly bathed in some antiseptic solution (five per cent. formalin preferably), and with a few fibres of cotton wrapped on a fine broach the canals being operated on are swabbed with the same solution.

The canals having been thoroughly opened and cleansed in this manner, it remains to treat them preparatory to dressing. This is done by first thoroughly washing them with three per cent. hydrogen dioxide and drying them with alcohol and hot air, when they are ready to receive the dressing. The smaller sizes of Swiss broaches, commonly known as jewellers' broaches, are ideal for dressing canals. The temper of these broaches is drawn and the ends squared by passing them through the pores of a short piece of hickory and sharpening on the oil-stone. A few fibres of unwaxed floss twisted on such a broach can be carried into a canal with the greatest facility. The square end catches the fibres of the silk and carries the dressing into the canal, while the polished surfaces of the broach permit of its being withdrawn, leaving the silk in position. In this manner a long fibre can be closely packed into a canal if desirable.

This is work that requires a high degree of skill, a delicacy of touch, and an intimate knowledge of the anatomy of the tooth, but, more than this, it requires infinite patience; indeed, patience is the first requirement, for without it it is quite impossible to attain unto either the skill or the knowledge. Our time should never be too valuable to give our best.

